# Expression Patterns of Cytokeratins (CK7, CK20, CK19, CK AE1/AE3) in Atypical Endometrial Hyperplasia Coexisting with Endometrial Cancer

**DOI:** 10.3390/ijms25169084

**Published:** 2024-08-21

**Authors:** Danuta Vasilevska, Vilius Rudaitis, Dorota Lewkowicz, Dominika Širvienė, Ugnius Mickys, Marek Semczuk, Bogdan Obrzut, Andrzej Semczuk

**Affiliations:** 1 Department of Gynecology, Vilnius University Hospital “Santaros Klinikos”, 08406 Vilnius, Lithuania; 2 Faculty of Medicine, Vilnius University, 03101 Vilnius, Lithuania; 3 Department of Clinical Pathology, Lublin Medical University, 20-090 Lublin, Poland; 4 National Centre of Pathology, Vilnius University Hospital “Santaros Klinikos”, 08406 Vilnius, Lithuania; ugnius.mickys@gmail.com; 5 Faculty of Medicine, Radom University, 26600 Radom, Poland; 6 Department of Obstetrics and Gynecology, Institute of Medical Sciences, Medical College, University of Rzeszow, 35-301 Rzeszow, Poland; bogdan.obrzut@gmail.com; 7 IInd Department of Gynecological Surgery and Gynecological Oncology, Lublin Medical University, 20090 Lublin, Poland

**Keywords:** cytokeratins, atypical endometrial hyperplasia, endometrial cancer, immunohistochemistry

## Abstract

Few studies have evaluated cytokeratin’s (CK) staining patterns in atypical endometrial hyperplasia (AEH) coexisting with early-stage endometrial cancer (EC). We aimed to assess the staining patterns of selected CKs (CK7, CK19, CK20, CK AE1/AE3) in 74 patients with coexisting AEH and EC by independently analyzing both morphological variables. Specimens were collected from women with AEH and EC who underwent surgical interventions between 2012 and 2019 at the Department of Obstetrics and Gynecology of Vilnius University Hospital “Santaros Klinikos” in Vilnius, Lithuania. Immunostaining was also qualitatively classified as being heterogeneous or intense. The results revealed heterogeneous CK7 expression in all AEH cases and intense staining in 95.95% cases of AEH. The heterogeneous expression of CK7 was detected in all EC specimens. Intense CK7 expression was observed in 95.09% cases of EC G1 and in all G2 ECs. Heterogenous CK19 expression was present in all AEH specimens with intense staining in 92.42% of cases. Heterogeneous CK19 expression was observed in all EC samples with intense expression in 86.27% cases of EC G1 and 100% cases of EC G2. Interestingly, a significant relationship was found when comparing the heterogeneous expression of CK19 between AEH and well-differentiated EC. A significant difference was reported in the intense expression of CK AE1/AE3 (*p* = 0.031; *p* = 0.029) between AEH and G2 ECs and in the intense expression of CK AE1/AE3 between G1 and G2 ECs. CK20 staining was not a characteristic feature for AEH and early-stage EC. CK staining is present either in AEH or in early-stage endometrioid-subtype EC in different manners. Heterogeneous CK19 expression was significantly more common in AEH than in EC. CK20 expression was not associated with either AEH nor early-stage EC. An intense expression of CK AE1/AE3 was mainly present in moderately differentiated ECs, whereas the intense reactivity of AE1/AE3 showed a significant difference in well to moderately differentiated uterine tumors. The clinical implication of CK staining may aid in the more accurate diagnosis of AEH and early-stage EC as well as detect micrometastases leading to better oncological outcomes.

## 1. Introduction

Currently, endometrial cancer (EC) is the most common gynecological malignancy in developed countries [1,2]. According to worldwide epidemiological statistical databa-ses, the incidence of EC is the 6th most common after breast, colon, lung, cervical, and thyroid cancer [1,2]. In 2020, 417.000 women were diagnosed with EC worldwide (standardized rate: 8.7/100.000 women) [2]. Numerous studies have demonstrated that EC is almost always preceded by endometrial hyperplasia (EH) [3]. Indeed, there is a 1–5% risk of simple EH progression to EC, whereas in case of atypical endometrial hyperplasia (AEH), the risk surges up to 29–54% [1,4,5]. According to the scientific literature, the coexistence of EC and EH occurs in 20–50% of cases [6,7,8,9,10,11,12].

Cytokeratins (CKs) are intermediate filament proteins that are preferentially expressed in epithelial tissues and play a critical role in maintaining epithelial cell integrity [13]. Currently, 28 genes of type I *CKs* (17 epithelial and 11 hair) and 26 genes of type II *CKs* (20 epithelial and 11 hair) have been identified. In the human genome, *CK* genes are expressed on two chromosomes: chromosome 17q21.2 (type I *CKs*, excluding *CK18*) and chromosome 12q13.13 (type II *CKs* and *CK18*) [13,14,15]. Epithelial carcinomas, as well as their distant metastases, usually express the same CKs as the healthy epithelium; thus, the detection of CKs is a valuable tool in determining the tumor origin. CKs are one of the most important molecular markers used in the diagnostics of oncological diseases. In laboratories worldwide, immunohistochemical tests are performed using specific CK antibodies [14]. Moll et al. (1983) were the first to report the expression of CK7, CK8, CK18, and CK19 in the ovarian mesothelium, epithelium of the fallopian tube, endometrium, and endocervix [14]. Tonofilaments of the vaginal squamous epithelium and exocervix showed positive expressions of CK4–CK6, CK13–16, and CK19, whereas ovarian tumors and EC tissue stained positive for CK7, CK8, CK18, and CK19, retaining the CK pattern of the healthy epithelium [16].

In the scientific literature, few studies have assessed the expression patterns of CKs in patients with AEH coexisting with early-stage ECs [17,18,19,20,21,22]. Moreover, the biological significance of localized CK expression patterns in the hyperplastic and neoplastic human endometrium has not yet been fully investigated.

The aim of the present study was to analyze the selected CK (CK7, CK19, CK20, CK AE1/AE3) expression patterns in 74 specimens of AEH and EC to independently analyze both morphological variables. Furthermore, the CK expression patterns of early-stage EC specimens were correlated with clinicopathological features.

## 2. Results

### 2.1. CK Expression in the Hyperplastic and Neoplastic Endometrium

CK immunostaining was performed in 74 patients with AEH concomitant with early-stage ECs. A summary of the expression patterns of CK7, CK19, and CK AE1/AE3 in precancerous and cancerous endometrial features is presented in Supplementary Table S1. The strongest CK20 expression in AEH and EC was weak enough to be hard to differentiate in all samples; hence, it is not included in the table.

Stained slides were assessed under a light microscope (Model SE, Nikon, Tokyo, Japan) at 400× magnification. We used a scale as follows: expression in less than 5% of the cells was evaluated as negative (0 points), expression in 6–25% of the cells was evaluated as weak (1 point), expression in 26–50% of the cells was evaluated as moderate (2 points), and expression in more than 50% of the cells was evaluated as strong (3 points) [22]. Immunostaining was also evaluated qualitatively as being heterogeneous or intense. The expression of CKs in AEH was compared to that in EC. In addition, the clinico-pathological features of early-stage EC were compared to the CK staining values. The intensity of CK expression was compared based on the correspondent distribution: 1p vs. 2p vs. 3p, 1p + 2p vs. 3p, and 1p vs. 2p + 3p.

**Figure 1:**
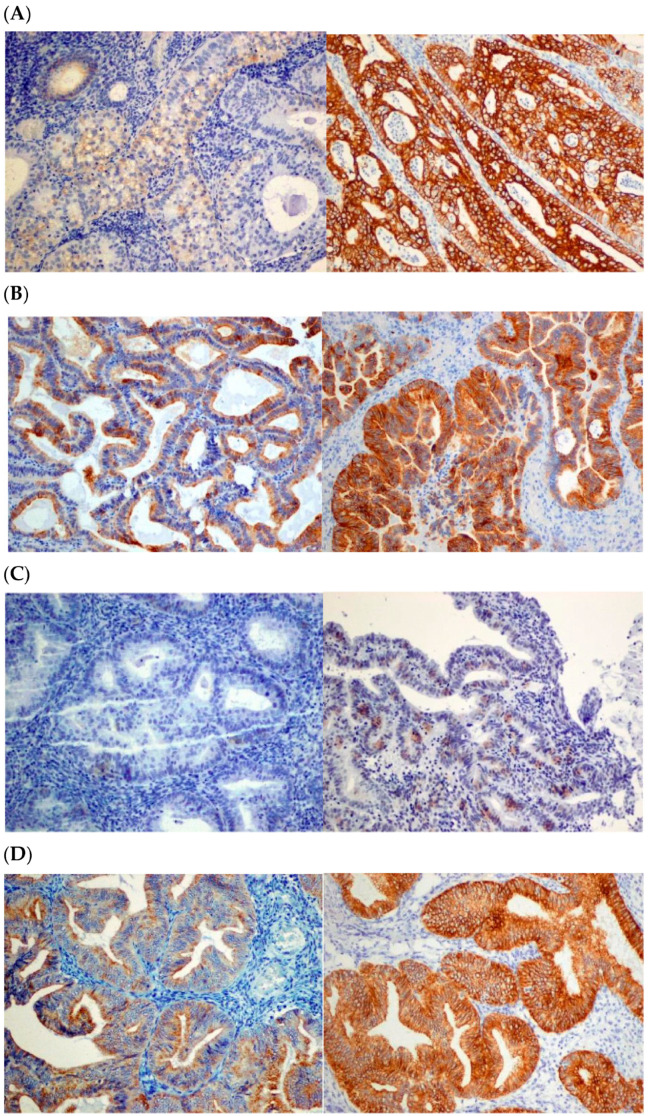
Weak (left column) and strong (right column) CK7 (**A**), CK19 (**B**), CK20 (**C**) and CK AE1/AE3 (**D**) immunostaining in atypical endometrial hyperplasia coexisting with EC (400×).

The weak and strong expression of selected CKs in AEH coexisting with EC samples is presented in Figure 1. Notably, CK staining was cytoplasmic in all cases investigated.

Otherwise, we found no significant difference between the expression of CK7 in AEH and EC nor when comparing AEH, EC G1, and G2 and when comparing G1 and G2 ECs. This finding indicates that CK7 is already expressed during the early stage of EC development.

### 2.2. Correlation of CK Staining between AEH and G1 EC

Significant differences were not reported when comparing the expression of CK7, CK19, and CK AE1/AE3 between AEH and G1 EC. Moreover, there was no significant difference in the expression of CK7, CK AE1/AE3, and intense expression of CK19 between AEH and well-differentiated EC. Interestingly, a significant relationship was found when comparing the heterogeneous expression of CK19 (1p vs. 2p vs. 3p; *p =* 0.04) between AEH and well-differentiated EC (Table 1).

### 2.3. Correlation of CK Staining between AEH and G2 EC

The expression of CK7 and CK19 and heterogeneous expression of CK AE1/AE3 showed no significant difference between AEH and G2 EC; however, a significant difference was reported in the intense expression of CK AE1/AE3 (1 vs. 2 vs. 3, *p* = 0.031; 1 + 2 vs. 3, *p* = 0.029) between AEH and EC G2 (Table 2 and Table 3).

### 2.4. Correlation of CK Staining between G1 and G2 ECs

No significant correlations were found in the expression of CK7 and CK19 and the heterogeneous expression of CK AE1/AE3 between EC G1 and G2. A significant relationship was detected in the intense expression of CK AE1/AE3 between EC G1 and G2 (comparing 1p vs. 2p vs. 3p, *p* = 0.007 and 1p + 2p vs. 3p, *p* = 0.008) (Table 4 and Table 5).

## 3. Discussion

According to the available data, the frequency of coexistence between AEH and EC is 10.3% to 48.5% [7,9,11,23,24,25,26,27,28,29,30,31,32,33,34]. The probability of EC occurrence after the diagnosis of AEH existed because of the inconsistency of diagnostic approaches among anatomopathologists and the clinicopathological variety of AEH recognition. Differentiation between AEH, which is a precursor of endometrioid-subtype EC, and uterine cancer is usually challenging [35]. For example, Zaino et al. (2006) assumed that the compatibility of pathologists’ AEH recognition is unacceptable, generally not exceeding 50% of the cases analyzed (38–47%) [36].

Specific CK monoclonal antibodies are widely applied to potentially serve as immunohistochemical markers in selected human malignancies [37]. Epithelial cancers retain the same CK expression pattern as primary healthy tissues. Moreover, CK profiling is especially valuable for poorly differentiated carcinomas spreading over several sites and for distant metastases of an unknown primary origin [37].

CK7 and CK19 expression is widespread in normal human epithelium; however, these CKs are not always co-expressed. In pathologic diagnosis, negative CK7 immunoreactivity is more valuable because most tumors are CK7-positive. 

The presented results revealed the heterogeneous expression of CK7 in all AEH specimens (81.54% of cases revealed moderate and strong expression), whereas intense expression was demonstrated in 95.95% of cases (55.41% of cases showed moderate and strong expression). The heterogeneous expression of CK7 was detected in all EC specimens, of which moderate and strong expression was reported in 89.09% of well-differentiated tumors and in 90.91% of moderately differentiated carcinomas. Intense CK7 expression was observed in 95.09% cases of EC G1 (moderate and strong-in 60.65%) and in all G2 uterine tumors (moderate and strong-in 46.15%). Interestingly, no previous study has compared the staining pattern of CK7 in human AEH coexisting with EC, highlighting the novelty of these findings.

The presented data are comparable with the results available from the scientific literature. For example, Chu et al. (2000) observed the expression of CK7 in the analyzed cases [18]. In a Canadian study, CK7 expression was reported in as many as 96% of endometrioid endometrial cancers and in all papillary-serous neoplasms [19]. In another report, CK7 positive staining was observed in all ECs analyzed with a mean staining grade of 8.5 (min: 4, max: 12) [20]. Park et al. (2009) reported that CK7 expression was positive in 100% of the evaluated specimens [38]. McCluggage 2006 highlighted that most cases of CK7 expression in carcinomas are detected particularly in uterine tumors [39]. In more recent data, CK7 expression was detected in 90.4% (206/228) of EEC cases, 93.3% (70/75) of serous EC cases, 53.8% (7/13) of EC G3 cases, and in 87.5% (7/8) of clear-cell carcinoma cases [21].

In the current study, heterogenous CK19 immunostaining was present in all specimens of AEH (81.48% moderate), whereas intense immunostaining was present in 92.42% of cases of AEH (60.6% moderate and strong). Heterogeneous CK19 expression was found in all EC samples, and moderate and strong expression was described in 67.5% of cases of EC G1 and in 76.92% of cases of EC G2. An intense expression of CK19 was observed in 86.27% of cases of EC G1 and 100% of cases of EC G2 with moderate and strong expression in 60.78% and 61.53% of the cases, respectively. Furthermore, immunohistochemical results proved that CK19 expression is present not only in the normal endometrium [17,40,41] but also in EC [38]. Another study assessed the expression of CK19 in the endometrium after the administration of norethisterone enanthate for 3–4 months, which was the same as in women with normal menstrual cycles. In contrast, CK19 expression was low in the endometrium of women who were administered Norplant [42]. Canadian scientists analyzed 66 cases of EC (endometrioid type n = 48, papillary-serous type n = 18) and concluded that CK19 was positive in 88% of EEC cases and in 94% of papillary-serous neoplasms [19]. In the present study, a significant difference was demonstrated between the expression of CK19 in AEH and well-differentiated EC (1p vs. 2p vs. 3p; *p* = 0.040). Interestingly, Stewart et al. (2011) investigated CK19 expression in 15 specimens of the proliferative (n = 5), secretory (n = 5), and atrophic (n = 5) endometrium and in 26 specimens of EEC G1 with the components of microcystic, elongated, and fragmented changes (MELF)-type invasion [43]. CK19 was expressed in all specimens of the normal endometrium; however, a more intense expression was noted in the functional layer, while the basal zone epithelium generally showed mild or even focal immunostaining. The proliferative epithelium was defined by stronger staining in the basal and apical cytoplasm. All cases of EEC showed CK19 expression, although the expression pattern differed in each tumor. Fifteen (58%) of the EEC specimens heterogeneously expressed CK19, whereas in the other 11 cases (42%), staining was only focal (from 10% to 25% of the neoplastic cells). In 21 cases of heterogeneous staining, only substantial malignant glands in the middle part of the tumor reacted strongly, whereas peripheral glands were characterized by weak-to-absent CK staining. In contrast, endometrioid carcinoma with areas of MELF-type glands showed homogeneous and considerable CK19 expression even when the rest of the tumor revealed no reactivity [43]. Because of the aforementioned facts, CK19 may be helpful in identifying single MELF-type cells beyond the tumor margins, lymphovascular space invasion (LVSI) and subtle lymph node metastases, which are characteristic of MELF-type EC. However, the role of CK19 reactivity is less clear in the setting of conventional EC. In summary, CK19 staining could be detected in the normal endometrium and EEC G1. However, malignancies usually stain focally in the central area of neoplastic glands or in infiltrative MELF-type glands. Therefore, further investigations are needed to determine the clinical significance of different CK19 staining patterns in particular types of uterine malignancies [43]. In a given study, the obtained results suggested that the expression of CK20 is not typical for AEH and EC cells. Similarly, Park et al. (2009) reported negative CK20 expression in the normal endometrium [38]. Numerous experimental studies have reported negative CK20 expression in EEC and papillary-serous EC [18,19,39]. In one such report, specimens were collected from 18 women diagnosed with EC and from 23 women who had undergone hysterectomy for benign reasons. The histological types of EC included a case of papillary-serous EC and 17 cases of EEC (seven cases-G1, 6-G2, 4-G3). It should be emphasized that all benign and malignant uterine diseases were CK20-negative [44]. In another investigation, Moll et al. (1992) concluded that a low expression of CK20 (<5%) was observed in 8 of 19 ECs, whereas CK20-negative staining was noted in normal endometria [45]. In another investigation, CK20 staining was positive in only 1 of 21 (4.8%) neoplasms [20]. Moreover, Park et al. (2009) reported that CK20 expression was observed in 3% (1/34) of EC specimens [38]. These results are comparable to those of other studies, where positive CK20 expression was reported in 1–5% cases of EC [46,47,48].

In terms of CK7+/CK20+, this profile was observed in all analyzed uterine cancer specimens [18]. Another study, assessing the CK7/CK20 profile, reported that CK7+/CK20+ was detected in 12% (3/25) specimens of EC, while CK7+/CK20− was detected in 80% (20/25), and CK7−/CK20− was detected only in 8% (2/25). None of the EC specimens showed the CK7/CK20+ profile [22]. A recent study by Dum et al. (2022) reported that CK20 expression was detected in 3.5% (8/231) of EEC cases but not in cases of serous EC (0/78), G3 EC (0/13) or clear-cell EC (0/8) [21].

AE1/AE3 is a broad-spectrum CK antibody cocktail that is commonly used in histopathologic assessments. AE1/AE3 consists of the mouse monoclonal antibody AE1, which recognizes acidic (type I) CKs 10, 14–16, and 19, and AE3, which reacts with basic (type II) CKs 1–8 [49]. The presented studies showed 100% heterogeneous expression of AE1/AE3 in AEH (56.98% moderate and strong), whereas intense expression was observed in 94.59% of AEH cases (81.08% moderate and strong). Heterogenous or intense CK AE1/AE3 expression was detected in all EC cases. Heterogeneous moderate and strong CK AE1/AE3 expression was noted in 64.28% of well-differentiated ECs and 90% of poorly differentiated uterine tumors, whereas intense moderate and strong expression was noted in 83.61% and 92.31% of specimens, respectively. Significant correlation was found when comparing the intense expression of CK AE1/AE3 between well- and moderately differentiated tumors (1p vs. 2p vs. 3p, *p* = 0.007; 1p + 2p vs. 3p, *p* = 0.008). Moreover, a significant difference was also demonstrated in the intense expression of CK AE1/AE3 (comparing 1p vs. 2p vs. 3p, *p* = 0.031; 1p + 2p vs. 3p, *p* = 0.029) between AEH and poorly differentiated uterine malignancies. These results are consistent with evidence from the scientific literature. For example, Wick et al. (1987) showed that CK AE1/AE3 was expressed in all 14 cases of EEC [50]. Canadian scientists investigated 73 cases of EC (endometrioid type n = 50, papillary-serous type n = 23), showing positive staining in 94% of EEC cases and in all cases of papillary-serous malignancies [19].

## 4. Materials and Methods

### 4.1. Patients and Tissue Samples

Seventy-four women who underwent surgical intervention between 2012 and 2019 in the Department of Obstetrics and Gynecology of Vilnius University Hospital “Santaros Klinikos” in Vilnius, Lithuania, were carefully selected. The main inclusion criterion was the coexistence on a slide of AEH with early-stage, endometrioid-type EC. Tissues were collected from women who were diagnosed with abnormal vaginal bleeding or from those who were diagnosed with abnormal endometrial thickness during an ultrasound scan, who then underwent surgery after the diagnosis of primary EC. The mean patient age was 56.5 (SD = 9.97; from 43 to 79 years). The clinical characteristics of the investigated patient group are depicted in Table 6.

The analyzed material was assessed based on clinicopathological parameters; histopathological cancer type and grade were assessed based on the revised WHO classification [51], whereas the clinical stage of the disease was determined according to the revised FIGO staging system [52]. In addition, the depth of myometrial invasion and LVSI were also analyzed. Histopathological analysis of the specimens was performed at the Department of Pathology, Vilnius University Hospital “Santaros Klinikos”, Vilnius, Lithuania (Table 7).

The patients were subdivided into two groups based on histopathological evaluation: endometrial intraepithelial neoplasia (EIN)/G1 (EIN/G1 pT1a + EIN/G1pT1b; n = 61) and EIN/G2 (EIN/G2pT1a; n = 13). Hematoxylin/eosin-stained slides were carefully re-examined by an experienced pathologist (DL) to confirm the diagnosis. No chemotherapy, hormonal therapy, or radiotherapy was administered to the patients before surgery. This research investigation was approved by the Independent Ethics Committee of the Vilnius University Hospital “Santaros Klinikos,” Vilnius, Lithuania (Kb reference No. 2020/6˗1237˗721).

### 4.2. Immunohistochemistry

Tissues obtained in the operation theater were immediately fixed in 10% buffered formalin (pH 7.4) and transported to the National Center of Pathology, Affiliate of Vilnius University Hospital Santaros Klinikos, where paraffin blocks were prepared according to a standardized laboratory procedure. Paraffin blocks were gently cut on 3 μm slides and placed on silanized slides (SIGMA, Lee on the Solent, Hampshire, UK). Slides were analyzed at the Department of Clinical Pathology, Lublin Medical University, Poland. Only specimens with confirmed coexisting AEH and EC were evaluated. Immunohistochemical staining was performed using the DAKO REAL EnVision/HRT kit (DAKO, North America Inc., CA, USA) according to the manufacturer’s instructions. We used primary antibodies from DAKO (North America Inc., CA, USA), the characteristics of which are presented in Table 8.

DAB (3.3′′-Diaminobenzidine tetrahydrochloride) was used as a chromogen. Following the reaction with DAB, the slides were counterstained with hematoxylin, dehydrated, and covered with a coverslip. Tissues positive for selected antibodies (based on the manufacturer’s instruction) were used as positive controls, and slides stained with normal serum, replacing the primary antibody, were used as negative controls.

### 4.3. Statistical Analyses

The Shapiro–Wilk test was used to test the hypothesis that the distribution of the data deviates from a comparable normal distribution. This distribution analysis was performed for all quantitative variables. In addition, for descriptive statistics, the mean value and standard deviation were applied, as all quantitative variables showed a normal distribution. A frequency table was created for qualitative valuables. Statistical comparison of groups was performed using Χ^2^ and Fisher’s exact tests. Statistical analysis was performed using the SPSS v. 17.0 (IBM SPSS Statistics, IBM Corporation, Chicago, IL, USA) statistical package and Microsoft Office Excel 2007. Statistical significance was defined when the *p*-value was <0.05.

## 5. Conclusions

On the basis of the conducted research, we concluded that CK staining is present either in AEH or in early-stage endometrioid-subtype EC in different manners. Interestingly, the heterogeneous expression of CK19 was significantly more frequently found in AEH than in EC. Moreover, CK20 expression was not associated with AEH or with early-stage EC. An intense expression of CK AE1/AE3 was present mainly in moderately differentiated ECs, whereas intense reactivity showed a significant difference when comparing well- to moderately differentiated uterine malignancies. According to the conducted research, CK7 and CK19 appear to be the most promising diagnostic and prognostic markers aiding conventional histopathological evaluation. In addition to more accurate AEH (EC precursor) detection, this immunohistochemical staining may be more sensitive for the identification of micrometastasis. CK staining of lymph nodes from EC patients has been proven to be more sensitive than traditional histopathologic evaluation for the detection of micrometastasis. More extensive investigation into the multifunctional role of CKs detection in neoplasms is anticipated for its more valid clinical significance.

## Figures and Tables

**Table 1:** Heterogeneous expression of CK19 in AEH and G1 EC.

Grade of Heterogeneous CK19 Expression	AEH	EC G1	*p*
1p	18.52%	32.5%	0.04 *
2p	81.48%	62.5%	
3p	0%	5%	

* Χ^2^ test.

**Table 2:** Intense expression of CKAE1/AE3 in atypical EH and moderately differentiated EC, comparing grades of expression: 1p vs. 2p vs. 3p.

Grade of Intense CKAE1/AE3 Expression	AEH	G2 EC	*p*
1p	13.51%	7.69%	0.031 *
2p	50.0%	92.31%	
3p	31.08%	0%	

* Χ^2^ test.

**Table 3:** Intense expression of CKAE1/AE3 in AEH and G2 EC, comparing grades of expression 1p + 2p vs. 3p.

Grade of Intensity CKAE1/AE3 Expression	AEH	EC G2	*p*
1p + 2p	63.51%	100%	0.029 *
3p	31.08%	0%	

* Fisher’s exact test.

**Table 4:** Intense expression of CK AE1/AE3 in G1 and G2 EC, comparing the grades of expression 1p vs. 2p vs. 3p.

Grade of Intensity CKAE1/AE3 Expression	EC G1	EC G2	*p*
1p	16.39%	7.69%	0.007 *
2p	47.54%	92.31%	
3p	36.07%	0%	

* Χ^2^ test.

**Table 5:** Intense expression of CK AE1/AE3 in G1 and G2 EC, comparing grades of expression 1p + 2p vs. 3p.

Grade of Intensity CKAE1/AE3 Expression	EC G1	EC G2	*p*
1p + 2p	63.93%	100%	0.008 *
3p	36.07%	0%	

* Fisher’s exact test.

**Table 6:** Clinical characteristics of the investigated patient group.

.	Mean (±SD)	Min-Max
Age (years)	56.63 (±9.97)	43–79
Number of pregnancies (n)	2.31 (±1.423)	0–5
Number of deliveries (n)	1.92 (±1.07)	0–5
Length of menopause (years)	6.96 (±9.351)	0–25
Body mass index (kg/m^2^)	31.31 (±8.147)	21.5–60.2

**Table 7:** Histopathological characteristics of the investigated specimens.

	Number of Cases n (%)
FIGO stage	
I	74 (100%)
Histological grade according to the WHO	
G1	61 (82.43%)
G2	13 (17.57%)
G3	0 (0%)
Myometrial invasion	
<1/2 of myometrium thickness	71 (95.95%)
≥1/2 of myometrium thickness	3 (4.05%)
LVSI	
Absent	74 (100%)

**Table 8:** Characteristics of the antibodies used in the experiments.

Antibody	Clone	Isotype	pH	Dilution
Cytokeratin 7	OV-TL 12/30	IgG1 kappa	High	1:50
Cytokeratin 19	RCK 108	IgG1 kappa	High	1:50
Cytokeratin 20	Ks20.8	IgG2a kappa	High	1:50
AE1/AE3	AE1/AE3	IgG1 kappa	High	1:100

## Data Availability

The data supporting reported results of this study are available upon reasonable request to the corresponding author.

## Supplementary Materials

The following supporting information can be downloaded at: https://www.mdpi.com/article/10.3390/ijms25169084/s1.
