# Supplementary material for: Expression Patterns of Cytokeratins (CK7, CK20, CK19, CK AE1/AE3) in Atypical Endometrial Hyperplasia Coexisting with Endometrial Cancer
Source: Int J Mol Sci. 2024 Aug 21;25(16):9084. doi: 10.3390/ijms25169084 (PMC11354644; doi:10.3390/ijms25169084)
Supplement: Supplementary file 1 [file ijms-25-09084-s001.zip › ijms-3101330-supplementary.pdf]

Table S1. Summary of CK7, CK19, and CK AE1/AE3 expression patterns in AEH corresponding to early-stage EC.

	CK expression	AEH	G1pT1a	G1pT1b	G1pT1	G2pT1a
**CK7**	Heterogenous 0p	0% (0/65)	0% (0/52)	0% (0/3)	0% (0/55)	0% (0/11)
	Intense 0p	4.05% (3/74)	5.17% (3/58)	0% (0/3)	4.91% (3/61)	0% (0/13)
	Heterogenous 1p	18.46% (12/65)	9.62% (5/52)	33.33% (1/3)	10.91% (6/55)	9.09% (1/11)
	Intense 1p	40.54% (30/74)	36.21% (21/58)	0% (0/3)	34.43% (21/61)	53.85% (7/13)
	Heterogenous 2p	63.08% (41/65)	65.38% (34/52)	33.33% (1/3)	63.64% (35/55)	81.82% (9/11)
	Intense 2p	39.19% (29/74)	44.83% (26/58)	100% (3/3)	47.54% (29/61)	30.77% (4/13)
	Heterogenous 3p	18.46% (12/65)	25.00% (13/52)	33.33% (1/3)	25.45% (14/55)	9.09% (1/11)
	Intense 3p	16.22% (12/74)	13.79% (8/58)	0% (0/3)	13.11% (8/61)	15.38% (2/13)
**CK19**	Heterogenous 0p	0% (0/54)	0% (0/38)	0% (0/2)	0% (0/40)	0% (0/13)
	Intense 0p	7.58% (5/66)	14.58% (7/48)	0% (0/3)	13.73% (7/51)	0% (0/13)
	Heterogenous 1p	18.52% (10/54)	34.21% (13/38)	0% (0/2)	32.5% (13/40)	23.08% (3/13)
	Intense 1p	31.82% (21/66)	25.00% (12/48)	33.33% (1/3)	25.49% (13/51)	38.46% (5/13)
	Heterogenous 2p	81.48% (44/54)	60.53% (23/38)	100% (2/2)	62.5% (25/40)	76.92% (10/13)
	Intense 2p	36.36% (24/66)	37.50% (18/48)	33.33% (1/3)	37.25% (19/51)	46.15% (6/13)
	Heterogenous 3p	0% (0/54)	5,26% (2/38)	0% (0/2)	5.0% (2/40)	0% (0/13)
	Intense 3p	24.24% (16/66)	22.92% (11/48)	33.33% (1/3)	23.53% (12/51)	15.38% (2/13)
**CK AE1/AE3**	Heterogenous 0p	0% (0/51)	0% (0/39)	0% (0/3)	0% (0/42)	0% (0/10)
	Intense 0p	5.41% (4/74)	0% (0/58)	0% (0/3)	0% (0/61)	0% (0/13)
	Heterogenous 1p	43.14% (22/51)	38.6% (15/39)	0% (0/3)	35.71% (15/42)	10.00% (1/10)
	Intense 1p	13.51% (10/74)	17.24% (10/58)	0% (0/3)	16.39% (10/61)	7.69% (1/13)
	Heterogenous 2p	49.14% (25/51)	51.28% (20/39)	100% (3/3)	54.76% (23/42)	80.00% (8/10)
	Intense 2p	50.0% (37/74)	48.28% (28/58)	33.33% (1/3)	47.54% (29/61)	92.31% (12/13)

	Heterogenous 3p	7.84% (4/51)	10.26% (4/39)	0% (0/3)	9.52% (4/42)	10.00% (1/10)
	Intense 3p	31.08% (23/74)	34.48% (20/58)	66.67% (2/3)	36.07% (22/61)	0% (0/13)
